# Dorsal root ganglion-targeted analgesic delivery for effective relief of neuropathic pain

**DOI:** 10.1016/j.mtbio.2025.102025

**Published:** 2025-06-26

**Authors:** Jiajia Sun, Jia Gu, Yan Ding, Xinyi Tu, Xiaohui Cai, Baochun Jiang, Zhongping Chen

**Affiliations:** a Institute of Special Environmental Medicine, Nantong University, Nantong, People's Republic of China; b Department of Anesthesiology, The Second Affiliated Hospital, Zhejiang University School of Medicine, Hangzhou, People's Republic of China; c Department of Hematology, The Third Affiliated Hospital of Nanjing Medical University, Changzhou, People's Republic of China; d Zhejiang Key Laboratory of Pain Perception and Neuromodulation, Hangzhou, People's Republic of China

**Keywords:** Neuropathic pain, Dorsal root ganglion, ADAM8, Targeted drug delivery, Lipid nanoparticles

## Abstract

Neuropathic pain is a devastating experience for patients and its treatment remains challenging. Dorsal root ganglion (DRG) is currently an important therapeutic target and DRG-targeted analgesic delivery through systemic injection is however not reported. Herein, a disintegrin and metalloproteinase protein 8 (ADAM8), a membrane-anchored protein primarily recognized as a cancer biomarker, is found to be de novo and persistently upregulated in the DRG neurons in spared nerve injury (SNI) and chemotherapy-induced neuropathic pain (CINP), two neuropathic pain models with distinct mechanisms. We thus designed a DRG-targeted delivery strategy using lipid nanoparticles (LNPs), aiming to effectively deliver conventional analgesics to the DRG to improve analgesic effect through blocking pain signal transduction from the periphery to central nervous system. *In vitro* and *in vivo* results revealed that LNPs extended the duration of action of the free analgesic from less than 6 h to more than 24 h and particularly, showed therapeutic superiority over conventional liposomes, achieved by their good structural stability for a more sustained release kinetics. After functionalized with a specific ADAM8 inhibitory peptide, the intravenously injected LNPs facilitated analgesic accumulation in the DRG in SNI and CINP. As a result, the LNPs significantly improved the intensity of action for pain relief in a single or repeated treatments, which ultimately relieved pain-related psychiatric comorbidities, while not causing latent systemic toxicities. To our best knowledge, it is the first example of nanoparticles-based DRG-targeted delivery strategy through systemic injection in treating neuropathic pain.

## Graphical abstract

**Figure:**
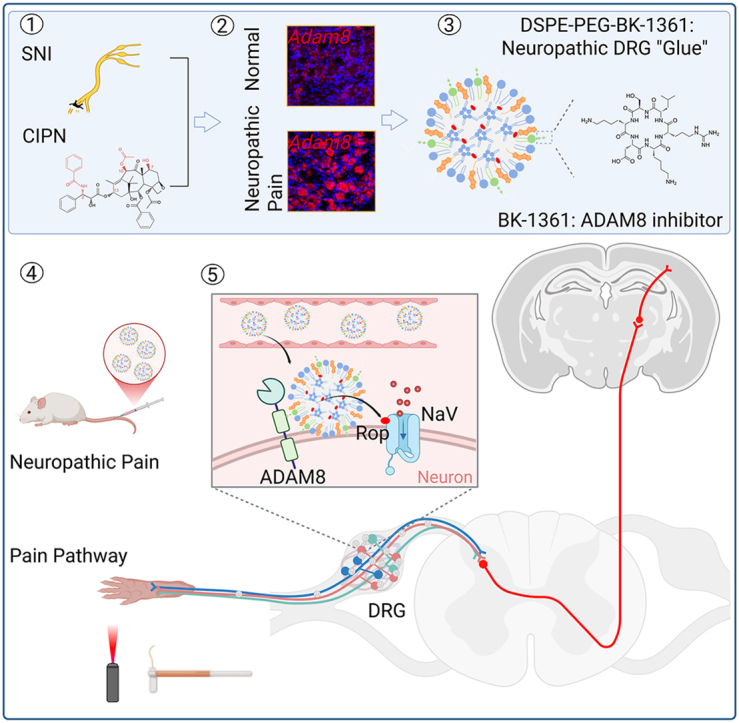

## 1 Introduction

Neuropathic pain is defined as a lesion or disease of the somatosensory and characterized by spontaneous ongoing or shooting pain, which can be evoked and amplified after noxious or non-noxious stimuli [1,2]. Unlike nociceptive pain, neuropathic pain is rather a consequence of pathological alterations than a symptom of a pathology and usually remains even after the pathology is treated. Neuropathic pain may originate peripherally (neuropathies, postherpetic neuralgia, etc.) or centrally (spinal cord injury, stroke, etc.), including nine conditions, as classified by the International Association for the Study of Pain [3]. Owing to the ageing global population, increased incidence of diabetes, and improved survival from cancer after treatment, the incidence of neuropathic pain is continuously increasing. Currently, neuropathic pain is the most recalcitrant chronic pain and affects up to 10 % of the general population [4,5]. Despite different mechanisms involved, the clinical manifestation of neuropathic pain is similar across diverse neuropathic syndromes and causes, which is also similar to other pain conditions. This not only makes it difficult to diagnose clinically, but also complicates treatment decisions, especially when it is in comorbid conditions. Together with limited treatment modalities, the treatment of neuropathic pain remains challenging. Impaired quality of life and associated other problems, such as function loss, anxiety, depression, disturbed sleep, and impaired cognition, result in neuropathic pain becoming a devastating experience for patients.

The management of neuropathic pain is currently centering around pharmacological treatments. First-line treatment is anticonvulsants and antidepressants for mild pain and second-line treatment includes local anaesthetics for moderate pain. When first- and second-line treatments are inefficient, strong opioids are used as third-line treatment for severe pain [5,6]. However, meta-analyses revealed that these treatments were effective in <50 % patients and achieved weak-moderate efficacy for 50 % of pain relief in these patients [7,8]. Lack of specificity, poor pharmacokinetics, and associated adverse effects even threatening life are the key factors for the dilemma. Progresses in the understanding of the pathophysiology of neuropathic pain are spurring the development of new targeted pharmacological treatments. A series of targeted molecules acting on ion channels, receptors tyrosine kinase, glutamatergic receptors, opioid receptors, and angiotensin II type 2 receptors are reported to inhibit pain [5,9]. Unfortunately, the translation of these targeted drugs has already been stagnant for decades and there are few successful cases, due to the dilemma in identifying specific neuropathic pain mechanisms in a given patient and hence selecting the targeted molecule most likely to be effective for this mechanism [10]. The pharmacological management of neuropathic pain is still confined to classic analgesics, aiming at pain relief.

In the past decades, nanomedicines using nanoparticles as carrier of marketed drugs have been well developed to treat diverse diseases, with more attention focused on cancer treatment. As nanomedicines can address some common critical issues of free drugs, such as poor pharmacokinetics and latent toxicity, they also open an avenue for pain management [[11], [12], [13]]. Up to date, there are approximately 60 approved nanomedicines, 5 of which are approved for pain relief [14,15]. However, although it is conclusive that like cancer nanomedicines, pain nanomedicines have better safety profiles than free drugs, their clinical efficacy and value are heavily doubted. Taking Exparel, a liposomal formulation of bupivacaine approved in 2021, as an example, only 6 of 36 studies found liposomal bupivacaine to be superior to plain bupivacaine [16]; peripheral nerve blockade was noted in only 8 of 25 volunteers receiving liposomal bupivacaine [17]. In addition, DepoDur, a liposomal formulation of morphine, failed to demonstrate superiority over plain morphine when used in low dose [18]. Pain nanomedicines are thus further surface-functionalized, enabling targeted delivery through binding with highly expressed pain receptors in painful sites, to improve analgesic effect. However, the case of such a strategy is quite few, due to the lack of pain biomarkers, and is limited to localized pain where pain source is identified [19]. In some types of neuropathic pain without an identified pain source and plausible biomarker, this strategy becomes not applicable.

In view that neuropathic pain can be perceived only when pain signals are transmitted to the cortex, regardless of its diversity in syndromes and mechanisms, pain transduction pathway-targeted delivery to block signal transduction is a more promising and universally applicable strategy for pain relief. The DRG is located between the dorsal root and spinal nerve, containing the greatest proportion of the body's sensory neurons. The DRG serves as a gateway for the transduction of sensory information from the periphery to central nervous system and the pathological changes in the DRG contribute to the initiation and persistence of neuropathic pain, especially following peripheral nerve injury [20]. DRG is currently an important target to treat neuropathic pain [[21], [22], [23]]. DRG-targeted pharmacological treatments include chemical targeting of ion channels, receptors, and signal pathways in the DRG through direct injection into the DRG, intrathecal injection, or epidural injection [24], which are technically challenging and seem to be limited to mechanistic study. DRG-targeted pain nanomedicines, enabling effective delivery of conventional analgesics to the DRG through systemic injection to improve analgesic effect and meet clinical requirement, are not reported yet.

**Scheme 1:**
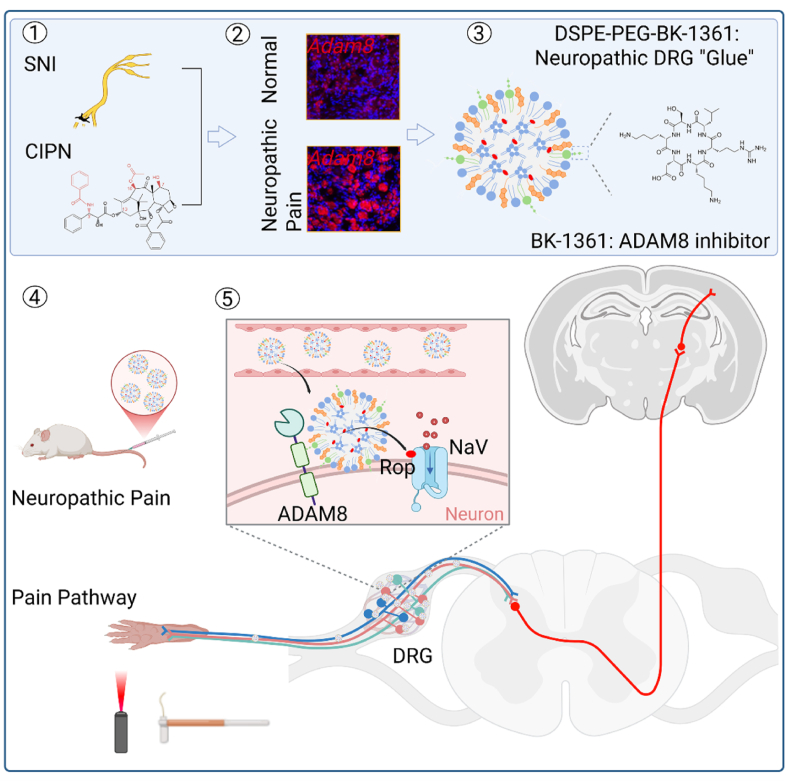
Schematic illustration of DRG-targeted analgesic delivery for effective relief of neuropathic pain. Spared nerve injury (SNI) and chemotherapy-induced neuropathic pain (CINP) modeling can lead to persistent upregulation of ADAM8 in neuronal cells of the DRG; BK-1361 conjugated lipid (DSPE-PEG-BK-1361) serving as neuropathic DRG “Glue” is incorporated into LNPs and following intravenous injection, the LNPs enable targeted analgesic delivery to the DRG through the binding affinity between BK-1361 and ADAM8; the accumulated analgesic in the DRG, using ropivacaine (Rop) as a model analgesic, is sustainedly released, acting on voltage-gated sodium (NaV) channel of neuronal cells for the blockage of pain signal transduction from the periphery to central nervous system, which ultimately extends the duration and intensity of action for effective relief of neuropathic pain. Image created in https://BioRender.com.

A disintegrin and metalloproteinase protein 8 (ADAM8) is an extracellular metalloproteinase, mainly regulating proteolysis and cell adherence. ADAM8 has typically low expression in normal tissues and is considered dispensable for normal development and homeostasis. On the other hand, ADAM8 is increased in a variety of cancer types, correlating with cancer progression, metastasis, chemoresistance, and poor prognosis, and primarily recognized as a cancer biomarker and a therapeutic target [25]. In this work, we first found that ADAM8 was persistently upregulated in the DRG neurons in two murine neuropathic pain models, indicating its deep involvement in the initiation and persistence of neuropathic pain. We thus surface-functionalized lipid nanoparticles (LNPs) with a specific ADAM8 inhibitory peptide (cyclo-RLsKDK, BK-1361), as the carrier of an analgesic, aiming to improve the effect of the analgesic in treating neuropathic pain through targeted delivery to the DRG with upregulated ADAM8 (Scheme 1).

## 2 Materials and methods

### 2.1 Chemicals

1,2-Dioleoyl-*sn*-glycero-3-phosphoethanolamine (DOPE), 1,2-distearoyl-*sn*-glycero-3-phosphoethanolamine-polyethylene glycol-2000 with and without terminated N-hydroxysuccinimide (DSPE-PEG-NHS and DSPE-PEG), and cholesterol were purchased from AVT Pharmaceutical Tech Co. Ltd (Shanghai, China). Paclitaxel (Ptx) and Rop were obtained from Aladdin Scientific Corp. (Shanghai, China). Lipofectamine 2000 and BK-1361, a cyclic peptide, were obtained from Glpbio (CA, USA). All other main chemicals were from Sinopharm Chemical Reagent Co. Ltd (Shanghai, China). All chemicals were used as received. Millipore water was used in all experiments unless otherwise indicated.

### 2.2 LNPs synthesis

#### 2.2.1 Synthesis of BK-1361 conjugated lipid

BK-1361 conjugated lipid was synthesized through the reaction between -NH_2_ of BK-1361 and active ester of DSPE-PEG-NHS according to the published works with some modifications [26,27]. Briefly, BK-1361 and DSPE-PEG-NHS (BK-1361: DSPE-PEG-NHS = 1/2, molar ratio) were mixed in anhydrous N,N-dimethylformamide (DMF) with magnetic stirring at room temperature for 10 h, followed by the addition of triethylamine (DSPE-PEG-NHS:TEA = 1/2, molar ratio). After another 36 h, the mixture was dialyzed against water (MWCO = 3500) for 24 h to generate BK-1361 conjugated lipid (DSPE-PEG-BK-1361). The structure of DSPE-PEG-BK-1361 was analyzed using a 400 MHz nuclear magnetic resonance (NMR) spectrometer (Avance III, Bruker).

#### 2.2.2 Synthesis of BK-1361 functionalized LNPs and drug loading

LNPs were synthesized based on our recent work [28], which was widely used to prepare vaccine-carrying LNPs [29]. In brief, DOPE, cholesterol, and DSPE-PEG-BK-1361 (7:2:1, molar ratio) were dissolved in anhydrous ethanol and then mixed with water (ethanol: water = 1/3, v/v). The resultant mixture was dialyzed against water (MWCO = 14000) for 3 h under gentle magnetic stirring to produce BK-1361 functionalized LNPs (BK-LNPs). Non-functionalized LNPs was also obtained using DSPE-PEG instead of DSPE-PEG-BK-1361.

Rop was mixed with all lipids in anhydrous ethanol to generate Rop-loaded LNPs (LNPs/Rop and BK-LNPs/Rop) according to the above procedure. The ratio of Rop to the lipids was 1/4 (w/w). With the same procedures, rhodamine B (RhB) loaded LNPs (LNPs/RhB and BK-LNPs/RhB) were also synthesized for fluorescence determination. All LNPs suspensions were lyophilized for further use.

### 2.3 Characterization

The size, morphology, polydispersity index (PDI), and zeta potential of LNPs were analyzed by a transmission electron microscopy (TEM, JEM-1230, Japan) or dynamic light scattering (DLS, Zetasizer ZS90, Malvern). Rop was quantified by high performance liquid chromatography (HPLC, Waters) to determine the entrapment efficiency and loading capacity of Rop in LNPs as well as *in vitro* Rop release from LNPs. Related information was detailed in Supporting materials.

### 2.4 Cell experiments

#### 2.4.1 Cell culture

Mouse neuroblastoma and rat DRG neuron hybrid cell line (ND7/23) was a gift from Zhejiang University. ND7/23 cells were cultured in Dulbecco's modified eagle's medium (DMEM) (37 °C, 5 % CO_2_ atmosphere), containing 10 % fetal bovine serum (FBS) and 1 % penicillin/streptomycin.

#### 2.4.2 Plasmid transfection

ADAM8 overexpression plasmid (ADAM8-OE) was also a gift from Zhejiang University. ND7/23 cells were seeded on a 6-well plate (5000/well) for 24 h. In the meanwhile, 2.5 μg of ADAM8-OE plasmid and 5 μL of lipofectamine 2000 were dispersed in 125 μL of serum-free Opti-MEN (Gibco), respectively, in two EP tubes for 5 min. The two mixtures were mixed, allowed to stand for 15 min, and then added to the cultured cells for transfection. After 48 h, the cells were collected for quantitative real-time polymerase chain reaction (qRT-PCR) to determine *Adam8* mRNA level or further treated to evaluate the targeting ability of BK-1361 to ADAM8.

#### 2.4.3 qRT-PCR

Total RNA was extracted from the transfected cells using TRIzol™ (ThermoFisher, MA) and reversed to cDNA by a transcription kit (Vazyme, Nanjing) according to the manufacturer's protocols. qRT-PCR was performed using SYBR Green PCR Master Mix (MCE, NJ) on a StepOnePlus system (ThermoFisher, MA). PCR run conditions were 5 min at 95 °C, 15 s at 95 °C, 30 s at 60 °C, and 15 s at 72 °C with 40 cycles. qRT-PCR primers were as follows: *Adam8* forward, TGGAGTCTGCCATGCTCTTG; *Adam8* reverse, TATACACGGAGGTCCTGGCA; *Actb* forward, CATCCGTAAAGACCTCTATGCCAAC; *Actb* reverse, ATGGAGCCACCGATCCACA. *Adam8* mRNA expression level was quantified by 2^−ΔΔCt^ method and normalized to *Actb*.

Similar protocols were used to determine *Adam8* mRNA expression level in the DRG as well as in some main organs.

#### 2.4.4 *In vitro* targeting ability evaluation

Cultured ND7/23 cells with or without ADAM8-OE were incubated with LNP/RhB and BK-LNPs/RhB for 6 h. Afterwards, the cells were fixed with paraformaldehyde (PFA), washed with PBS (pH = 7.4) twice, and stained with DAPI. Finally, the fluorescence of the cells was captured on a confocal laser scanning microscope (CLSM, TCS SP8, Leica) using DAPI and RhB-related filters. Captured images were analyzed by ImageJ software.

#### 2.4.5 Cell viability

Cell viability was assessed by MTT assay. ND7/23 cells with or without ADAM8-OE were seeded in a 96-well plate and then incubated with Rop, LNPs/Rop, and BK-LNPs/Rop at a fixed Rop concentration (100 μg/mL). Following 48 h incubation, the cells were washed and treated for MTT assay on a microplate reader (Synergy 2, BioTek).

### 2.5 Animal experiments

#### 2.5.1 Animals

Animal experiments were carried out on ICR mice. ICR mice (6–8 weeks old, male) were supplied by Animal Center of Nantong University. All animal experiments were approved by the Animal Care and Use Committee of Nantong University (Approval ID: S20241202-002) and the Jiangsu Province Animal Care Ethics Committee (Approval ID: SYXK[SU] 2007–0021). All animals were maintained in controlled conditions: 22 ± 1 °C at room temperature, 55 ± 10 % in humidity, 12 h light/dark cycle, and free access to food and water.

#### 2.5.2 Spared nerve injury (SNI)-induced neuropathic pain model

SNI surgery was performed as previously described to induce peripheral neuropathic pain [30]. Briefly, under 3 % isoflurane anesthesia via inhalation, one of the hind limbs of ICR mice was incised to expose three branches (common peroneal, tibial, and sural nerves) of the sciatic nerve. While the sural nerve was left intact, the common peroneal and tibial nerves were carefully ligated with 6-0 silk. The wound was sutured and the mice were returned to housing after their quick recovery. A sham group was operated as described above except for ligation.

#### 2.5.3 Chemotherapy-induced neuropathic pain (CINP) model

CINP was generated according to a reported work [31]. A Ptx stock solution was prepared by dissolving Ptx powder in a solution of 4 % dimethyl sulfoxide and 4 % Tween-80 in sterile PBS (pH = 7.4) at a concentration of 1 mg/mL. This stock solution was intraperitoneally injected into BALB/c mice at a dose of 2 mg PTX/kg body weight every two days for a cumulative dose of 8 mg/kg in 6 days. All mice became hyperalgesic after the treatments.

#### 2.5.4 DRG immunofluorescence and *in vivo* targeting ability evaluation

SNI and CINP mice were injected with LNP/RhB and BK-LNPs/RhB either intrathecally or intravenously. At 6 h post-injection, the mice were euthanized through cervical dislocation and subjected to perfusion. The L4-L6 DRG was collected, fixed in 4 % PFA overnight, gradually dehydrated with 20 % and 30 % sucrose, and embedded into OCT for cutting on a freezing microtome. The obtained DRG sections (14 μm in thickness) were blocked with 10 % BSA for 1 h and then sequentially incubated with neuronal class III β-tubulin (Tuj1) primary antibody (D71G9, rabbit, Cell Signaling Technology, #5568, 1:1000) overnight and its fluorescence-labeled secondary antibody (Alexa Fluor 488 anti-rabbit IgG, donkey, ThermoFisher, #A-21206, 1:500) for 2 h. The fluorescence of Tuj1 and RhB was captured on the SP8 CLSM using the corresponding filters to evaluate the targeting ability of BK-1361.

#### 2.5.5 RNAscope

RNA-scope assay was performed for in situ evaluation of *Adam8* (Advanced Cell Diagnostics, #1047421-C1) in the DRG of the mice using RNA-Scope 2.5 HD Red Kit (Advanced Cell Diagnostics, #322360) following our recent work [32,33]. In detail, the fixed DRG were sectioned into 14 μm slices and underwent baking at 60 °C for 1 h, dehydration in 100 % ethanol, pretreatment with hydrogen peroxide and protease digestion, probe hybridization, signal amplification, chromogenic development using Fast Red, DAPI counterstaining and mounting. Images were acquired using a Leica Thunder imaging system (DM6B, Leica).

#### 2.5.6 Plasma drug concentration and tissue distribution

Plasma drug concentration and tissue distribution of Rop in different formulations were investigated in SNI mice. Mice were intravenously injected with free Rop, LNPs/Rop, and BK-LNPs/Rop at a dose of 3 mg/kg (n = 5) once. For plasma drug concentration, the mice were euthanized to collect blood at 1 min, 10 min, 1 h, 2 h, and 6 h post-injection. For tissue distribution, the heart, brain, liver, spleen, lung, and spleen were harvested at 6 h post-injection. Collected blood and tissue samples were treated for Rop quantification by HPLC with detailed protocols described in Supporting materials.

#### 2.5.7 Grouping and drug treatment

For SNI mice, mice were randomly divided into 5 groups: one control group (PBS), one free drug group (free Rop), one drug-free blank LNPs group (BK-LNPs), and two drug-loaded LNPs formulation groups (LNPs/Rop and BK-LNPs/Rop) (n = 8), receiving intravenous injection on the 7th, 14th, and 21st days following modeling. LNPs/Rop group was set to demonstrate that LNPs could extend the duration of action of free Rop; BK-LNPs/Rop group was set to demonstrate BK-LNPs could further improve the intensity of action through DRG-targeted delivery; BK-LNPs group was set to verify that the improved intensity of action of BK-LNPs/Rop was owing to DRG-targeted delivery of Rop rather than BK-1361 modification, as BK-1361 alone showed a certain analgesic effect (data not shown). In another experiment, SNI mice were randomly divided into the following 3 groups: free Rop, LNPs/Rop, and BK-LNPs/Rop (n = 8), receiving intrathecal injection once on 7th day following modeling.

For CINP mice, mice were randomly divided into 4 groups: PBS, Rop, LNPs/Rop, and BK-LNPs/Rop (n = 8), receiving intravenous injection on the 7th and 21st days following modeling. The dose of Rop was 3 mg/kg for intravenous injection, while it was 1 mg/kg for intrathecal injection. Rop concentration in different formulations for injection was around 0.2 % and each injection was completed in 30 s.

#### 2.5.8 Neuropathic pain test

Neuropathic pain was tested by measuring mechanical allodynia and thermal hyperalgesia of mice. Mechanical allodynia was determined using Frey filaments pioneered by Dixon and thermal hyperalgesia was examined through Hargreaves's test [34,35]. Paw withdrawal threshold and withdrawal latency in responding to stimuli were determined to assess neuropathic pain. Detailed protocols were described in our recent works [28,36]. The test was performed at 12, 24, and 48 h following each injection.

#### 2.5.9 Elevated plus maze (EPM) and forced swim test (FST)

EPM and FST were conducted to determine anxiety- and depression-like behaviors, respectively, in SNI mice receiving intravenous injection on the 7th, 14th, and 21st days following modeling, as described in Section 2.5.6. Both EPM and FST were conducted on the 25th day. For EPM, mice were placed in the middle of the four-arms junction of a maze (35 cm × 5 cm) and allowed to explore the maze for 5 min. The trajectory of the mice was recorded to determine the percentage of time spent in the open arms relative to total time [37]. For FST, mice were placed into a plexiglass cylinder (46 cm × 20 cm) with warm water (25 °C). A mounted camera was employed to record the duration of immobility [38]. Mice were placed into the same conditions for acclimatization one day in advance.

#### 2.5.10 Hematologic study and histopathological analysis

Hematologic study was carried out in SNI mice for biosafety assessment. Following EPM and FST, all mice were sacrificed and blood was collected for hematologic study. Serum aspartate aminotransferase (AST), lactate dehydrogenase (LDH), creatine kinase (CK), albumin (ALB), alanine aminotransferase (ALT), blood urea nitrogen (BUN), and creatinine (CR) were recorded on a Biochemical Auto-analyzer (Roche, USA). Meanwhile, the heart, brain, liver, spleen, lung, and kidney were harvested, fixed with PFA, embedded in paraffin, and sectioned for hematoxylin-eosin (H&E) staining. All sections were imaged on a Leica DM4000B microscope.

### 2.6 Statistical analysis

Data were expressed as mean ± standard deviation (SD) or mean ± standard error of the mean (SEM) and analyzed using GraphPad Prism 8.0. Unpaired student's *t*-test and one-way analysis of variance (ANOVA) test were used to compare two groups and multiple groups, respectively. A statistical significance was set at *p* < 0.05.

## 3 Results and discussion

### 3.1 Synthesis, characterization, and *in vitro* targeting ability evaluation

Liposomes are the most commercially successful drug delivery system used in the management of several high-risk diseases. To date, there are 14 liposomal nanomedicines, accounting for 25 % of approved nanomedicines, and 40 % of approved pain nanomedicines are liposomes (2/5) [14]. LNPs, made of lipids but structurally different from liposomes, have been demonstrating the value and rapid translational potential, due to their great success in vaccine delivery to combat COVID-19. Particularly, we first reported that LNPs as carriers of chemotherapeutic agent could overcome some drawbacks of liposomes, such as drug leakage and unsatisfactory pharmacokinetics, to maximize therapeutic potency and showed therapeutic superiority over liposomes [28]. LNPs are therefore chosen as the carrier of pain nanomedicine in this work. On the other hand, the selection of analgesics puts us in a dilemma and Rop, a representative local anesthetic is finally chosen. The reason will be extensively discussed in the safety assessment section. It is hypothesized that while reducing toxicity, LNPs can effectively extend the duration of Rop for long-acting analgesia and surface-functionalizing LNPs for DRG-targeted delivery can further improve analgesic effect.

**Fig. 1:**
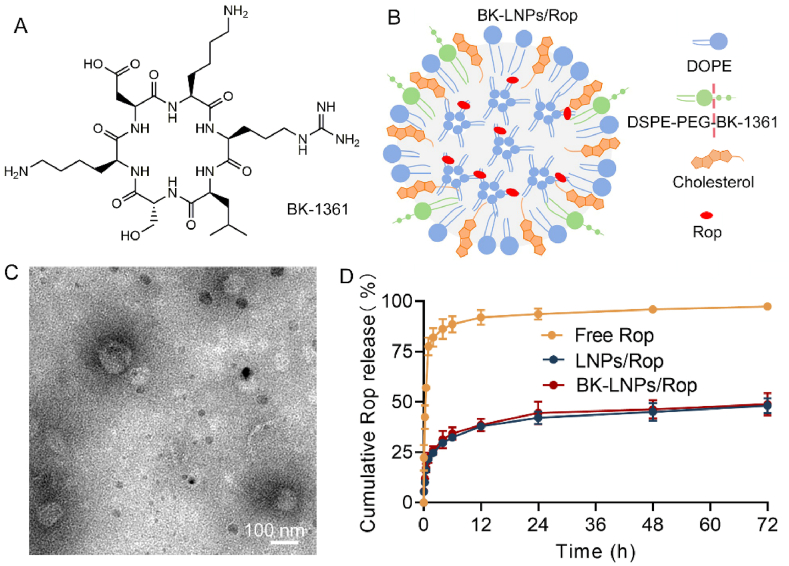
Structure and characterization of LNPs. (A) Structure of BK-1361. (B) Schematic illustration of suggested structure of BK-LNPs/Rop. (C) TEM image of BK-LNPs/Rop. Blank dots in TEM image should be crystallized phosphotungstic acid used for negative staining. (D) Cumulative drug release of free Rop, LNPs/Rop, and BK-LNPs/Rop within 72 h. Data were expressed as mean ± SD (n = 3).

By mimicking the RLSKDK motif of ADAM8, a series of six amino acids cyclic peptides, whose peptide sequence is modified with D-form amino acids in place of arginine, leucine, and serine, are generated to prevent ADAM8 multimerization. Among them, BK-1361 with the peptide sequence containing a D-serine (RLsKDK, Fig. 1A) shows particularly high selectivity toward ADAM8 [39] and is hence selected as targeting moiety to endow LNPs with DRG-targeting ability. BK-1361 was first conjugated with DSPE-PEG-NHS to produce DSPE-PEG-BK-1361 with the structure confirmed by ^1^H NMR spectra. As shown in Fig. S1, while the proton peak of -CH_2_ from PEG around 3.50 ppm remained unchanged, the area of the proton peak of -CH_3_ at 0.85 ppm from DSPE and BK-1361 increased, indicating the successful conjugation. The target lipid was then incorporated into LNPs to form BK-LNPs. Compared with liposomes with the structure being clearly depicted, the structure of LNPs remains unclear and controversial, which might vary with synthetic protocol, molecular structure of lipid, lipid composition, and type of payload. It is generally conjectured that LNPs have multiple inverted micelle-like lipid compartments encompassed by a lipid monolayer, enabling the loading of both hydrophobic and hydrophilic species (Fig. 1B) [40,41]. DLS results of BK-LNPs as well as LNPs with or without drug loading were summarized in Table S1 and Fig. S2. All LNPs exhibited 140–165 nm in hydrodynamic size with negative surface charge below −23.0 mV. Drug loading exerted no considerable influence on their size, PDI, and surface charge. HPLC revealed that LNPs and BK-LNPs achieved a high drug entrapment efficiency (>85.0 %) for a high loading capacity (>17.5 %) (Table S2), as a result of a high percentage of hydrophobic inner for loading hydrophobic Rop. A presentative TEM image of BK-LNPs/Rop was presented in Fig. 1C, indicating the spherical morphology with an average size less than 100 nm in their dried and shrunk state. As expected, LNPs had better structural stability for Rop retaining than liposomes, as verified by cumulative release profiles (Fig. S3), which would ultimately benefit the duration of analgesic effect; importantly, BK-LNPs displayed very similar release dynamics to LNPs (Fig. 1D), suggesting that surface functionalization with BK-1361 did not impair the stability of LNPs. The release curves also demonstrated that LNPs could keep stable within at least 72 h, as approximately 50 % of Rop was released, which approached a transmembrane balance of payload.

**Fig. 2:**
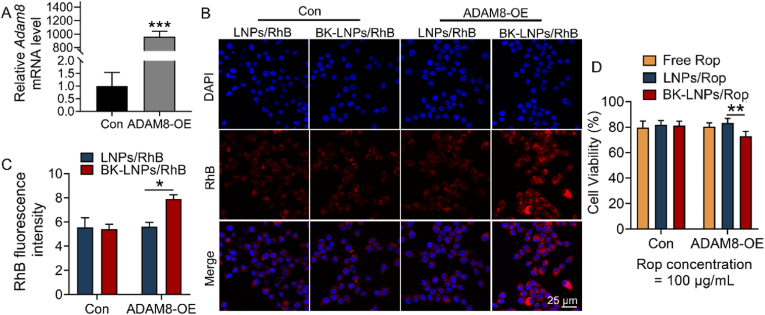
*In vitro* targeting ability and cell viability evaluation. (A) Relative *Adam8* mRNA level of ND7/23 cells with or without ADAM8-OE, determined by qRT-PCR. (B) CLSM images of ND7/23 cells treated with LNPs/RhB and BK-LNPs/RhB and (C) corresponding quantitative analysis of RhB fluorescence. (D) Cell viability of ND7/23 cells treated with free Rop, LNPs/Rop and BK-LNPs/Rop, determined by MTT. Data were expressed as mean ± SD (n = 3 for A and C; n = 6 for D). ∗*p* < 0.05, ∗∗*p* < 0.01, and ∗∗∗*p* < 0.001.

*In vitro* targeting ability evaluation of BK-LNPs was studied by comparing their cell uptake with LNPs, which was performed on ND7/23 cells. As ND7/23 cells themselves had low ADAM8 expression, they were transfected with ADAM8-OE, leading to a significant increase in mRNA level (Fig. 2A). It is worth noting that as available antibody specific to ADAM8 protein is unfortunately not found, ADAM8 expression is only determined by qRT-PCR. In the following, the cells were treated with LNPs/RhB and BK-LNPs/RhB, and cell uptake was studied by determining RhB fluorescence. As shown in Fig. 2B and C, the uptake of LNPs and BK-LNPs in the cells without ADAM8-OE was similar; in comparison, the uptake of BK-LNPs in the cells with ADAM8-OE was dramatically elevated, solidly corroborating the targeting affinity between BK-LNPs and ADAM8-overexpressed cells. Cell viability by MTT revealed that Rop was relatively less toxic and at the concentration of 100 μg/mL, the cells treated with free Rop and LNPs/Rop remained approximately 80 % viability, irrespective of ADAM8-OE transfection. The toxicity of BK-LNPs/Rop for the cells without ADAM8-OE was similar to LNPs/Rop, which was however significantly augmented for the cells with ADAM8-OE (Fig. 4D). The augmented toxicity of BK-LNPs/Rop should be not merely owing to improved Rop uptake through targeting affinity, but rather a synergistic effect of improved uptake and the inhibitory potency of BK-1361 on cells with ADAM8 overexpression. This result also suggests that BK-LNPs as drug carrier are safe for normal cells and tissues, as they originally have low ADAM8 expression.

### 3.2 BK-LNPs enable DRG-targeted delivery in SNI and CINP

**Fig. 3:**
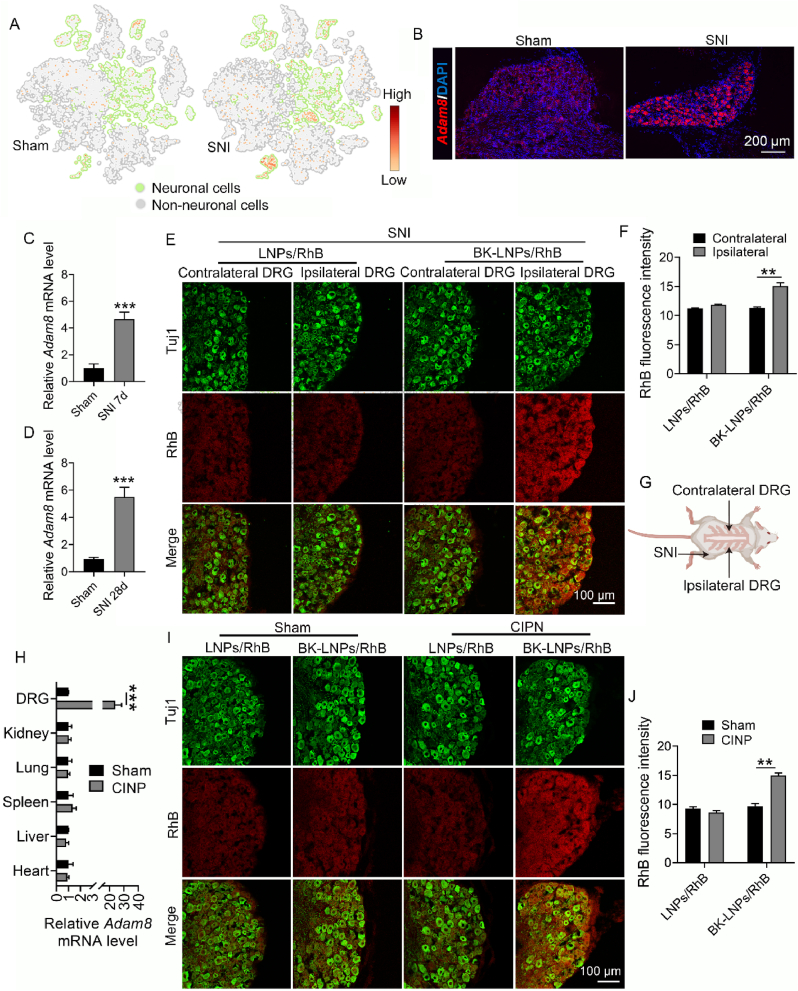
*In vivo* DRG-targeting ability of BK-LNPs in SNI and CINP. (A) t-distributed stochastic neighbor embedding (t-SNE) visualization of *Adam8* mRNA expression patterns across DRG cellular subpopulations and (B) RNAscope imaging for *Adam8* mRNA in the DRG neurons following SNI modeling. (C–D) Relative *Adam8* mRNA expression level in the DRG on the 7th and 28th days following SNI modeling. (E) CLSM images of the bilateral DRGs ipsilateral and contralateral to SNI surgical site and (F) corresponding quantitative analysis of RhB fluorescence. Tuj1 was a neuronal marker. (G) Schematic illustration for the bilateral DRGs ipsilateral and contralateral to SNI surgical site. (H) Relative *Adam8* mRNA expression level in the DRG as well as in the heart, liver, spleen, lung, and kidney on the 7th day following CINP modeling. (I) CLSM images of the DRG in CINP and (J) corresponding quantitative analysis of RhB fluorescence. For CLSM imaging, mice were intravenously injected with LNP/RhB and BK-LNPs/RhB and at 6 h post-injection, mice were euthanized for analysis. Data were expressed as mean ± SEM (n = 3 for C and D; n = 5 for F, H, and J). ∗∗*p* < 0.01 and ∗∗∗*p* < 0.001.

*In vivo* DRG-targeting ability of BK-LNPs was first studied in SNI, an easy-to-operate model producing reproducible, robust, and long-lasting neuropathic pain through the ligation of the common peroneal and tibial nerves of the hind limb [42,43]. As shown in Fig. 3A, single-nucleus RNA-sequencing data revealed a substantial upregulation of *Adam8* mRNA predominantly in DRG sensory neurons following SNI modeling. This finding was further corroborated by RNAscope experiments (Fig. 3B). *Adam8* mRNA expression in the DRG was also determined by qRT-PCR. It was observed that *Adam8* mRNA in the total DRG was significantly elevated after 7 days following SNI modeling, which persisted for at least 28 days (Fig. 3C and D). In fact, SNI modeling only considerably upregulated *Adam8* mRNA in the DRG ipsilateral to SNI surgical site, while not affected the contralateral DRG (data not shown). In short, these results provided the prerequisite for DRG-targeted delivery of BK-LNPs. Considering that local anaesthetics are usually used in regional, intrathecal, or epidural routes, BK-LNPs/RhB as well as LNPs/RhB were intrathecally injected and their distribution in the DRGs ipsilateral and contralateral to SNI surgical site was imaged. Unfortunately, BK-LNPs/RhB displayed no significant difference between the bilateral DRGs; there was also no difference found between BK-LNPs/RhB and LNPs/RhB (Fig. S4). This unexpected phenomenon should be largely due to confined drug diffusion through intrathecal injection. As systemic administration of local anesthetics is increasingly suggested to provide clinical analgesia in a broad range of neuropathic pain states, intravenous injection, the first-line systemic route of administration for nanomedicines including pain nanomedicines [44], was utilized for experimental proofs of concept of DRG-targeting ability of BK-LNPs. While there was no difference found between the bilateral DRGs of LNPs/RhB group following intravenous injection, the distribution of BK-LNPs in the DRG ipsilateral to SNI surgical site considerably increased compared with that in the contralateral DRG (Fig. 3E–G), realizing DRG-targeted delivery.

**Fig. 4:**
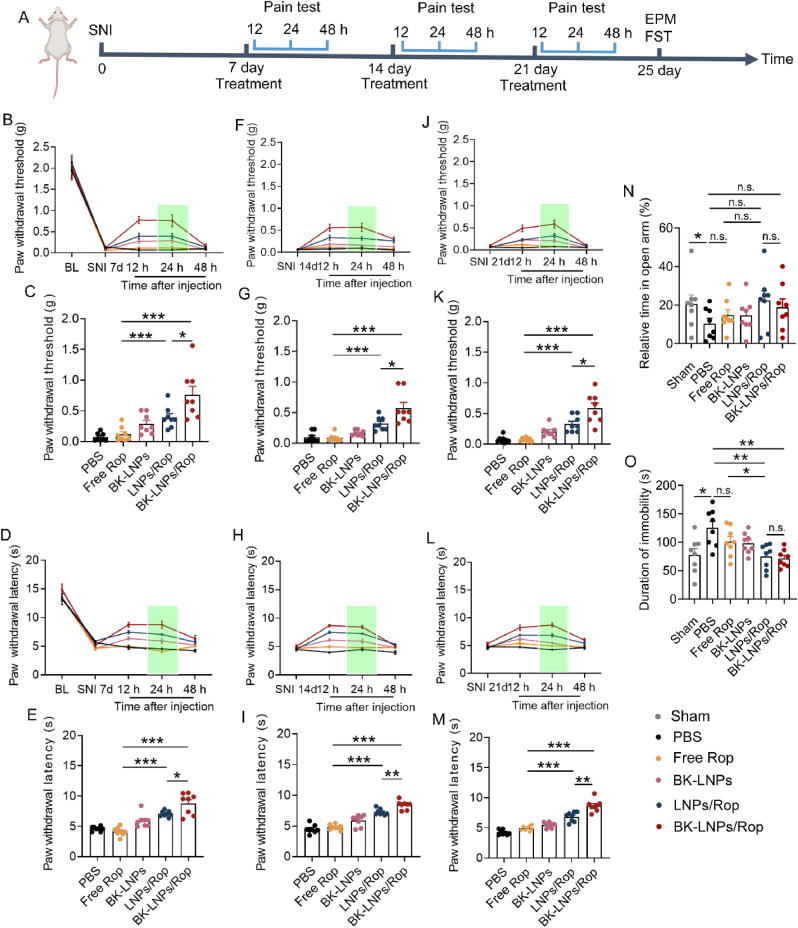
*In vivo* evaluation of neuropathic pain and its resultant anxiety- and depression-like behaviors in SNI. (A) Protocol of drug treatment for behavioral test. (B) Mechanical allodynia and (D) thermal hyperalgesia examination at 12 h, 24 h, and 48 h following the injection on the 7th day and (C and E) corresponding analysis at 24 h. (F) Mechanical allodynia and (H) thermal hyperalgesia examination at 12 h, 24 h, and 48 h following the injection on the 14th day and (G and I) corresponding analysis at 24 h. (J) Mechanical allodynia and (L) thermal hyperalgesia examination at 12 h, 24 h, and 48 h following the injection on the 21st day and (K and M) corresponding analysis at 24 h. (N) EPM and (O) FST for anxiety- and depression-like behavior evaluation, respectively, conducted on the 25th day. Mice received intravenous injection of PBS, free Rop, LNPs/Rop, BK-LNPs, and BK-LNPs/Rop at 3 mg Rop/kg body weight. BL data came from healthy mice before SNI modeling. Data were expressed as mean ± SEM (n = 8). ∗*p* < 0.05, ∗∗*p* < 0.01 and ∗∗∗*p* < 0.001. n.s. = not significant.

DRG-targeting ability was further studied in CINP. CINP is an adverse outcome of chemotherapy associated with a broad spectrum of chemotherapeutic agents as well as their dose, intensity, and duration, with the mechanism not fully understood yet [45]. Particularly, it affects up to 90 % of patients treated with taxanes, platinum compounds, and vinca alkaloids [46]. ADAM8 is also found involved in CINP. As shown in Fig. 3H and Fig. S5, *Adam8* mRNA expression in the DRG was significantly elevated on the 7th and 21st days following CINP modeling. Moreover, the expression in the heart, liver, spleen, lung, and kidney remained unchanged, affirming that CINP but not Ptx itself contributed to ADAM8 upregulation in the DRG. The upregulated ADAM8 also realized DRG-targeted delivery in CINP, as verified by significantly increased distribution of BK-LNPs in ADAM8-upregulated DRG (Fig. 3I and J). Generally, the results in two neuropathic pain models strongly suggest the deep involvement of ADAM8 in the DRG, especially in peripheral neuropathic pain, which might be independent of specific cause of neuropathic pain. Using ADAM8 as the target, DRG-targeted delivery can be achieved. As systemic injection of local anaesthetics is proved efficacious in relieving neuropathic pain [47], DRG-targeted Rop delivery using BK-LNPs is believed to improve analgesic effect, which is our focus in the following.

### 3.3 DRG-targeted analgesic delivery effectively relieves neuropathic pain and psychiatric comorbidities in SNI

Neuropathic pain manifests with diverse symptoms, each of which is mediated by a distinct mechanism. Allodynia, a pain elicited by a stimulus not causing pain, and hyperalgesia, an increased pain evoked by a stimulus that usually causes pain, are the two particularly prominent symptoms in different types of neuropathic pain [48,49]. In this work, SNI mice were intravenously treated and their neuropathic pain was assessed by determining mechanical allodynia and thermal hyperalgesia, which were also applicable to patients with suspected neuropathic pain [50]. The detailed protocols were illustrated in Fig. 4A. Mechanical allodynia results revealed that the baseline (BL) of paw withdrawal threshold of mice prior to SNI modeling was around 2.0 g and severe mechanical allodynia was observed with the threshold sharply decreasing to approximately zero on the 7th day following SNI modeling (Fig. 4B). This result was also indicative of the success of neuropathic pain modeling. Free Rop did not exert any effect on mechanical allodynia at 12 and 24 h post-injection. Loading Rop into LNPs not only protected it against rapid metabolism but also enabled sustained release, both of which would benefit the duration of action. As a result, LNPs/Rop remarkably relieved mechanical allodynia and extended the duration with elevated paw withdrawal threshold lasting at least 24 h. BK-LNPs/Rop further significantly relieved mechanical allodynia compared with LNPs/Rop (∗*p* < 0.05) (Fig. 4C). A similar trend was observed in thermal hyperalgesia examination (Fig. 4D). The BL of paw withdrawal latency was around 15 s, which decreased to around 5 s on the 7th day following SNI modeling. LNPs/Rop effectively relieved thermal hyperalgesia and extended the duration; BK-LNPs/Rop further significantly improved the potency compared with LNPs/Rop (∗*p* < 0.05) (Fig. 4E). HPLC result validated that BK-LNPs facilitated Rop distribution to ADAM8-upregulated DRG ipsilateral to SNI surgical site for analgesia improvement (Fig. S6), which was in good consistency with the fluorescence results (Fig. 3E and F).

The analgesic effect of free Rop, LNPs/Rop, and BK-LNPs/Rop in a short period following the injection was also studied. As shown in Fig. S7, free Rop actually displayed analgesic effect at 2 h post-injection, which was better than LNPs/Rop and BK-LNPs/Rop at this timepoint; however, its effect decreased over time, resulting from its rapid metabolism and leading to the duration of action less than 6 h. In contrast, the analgesic effect of LNPs/Rop and BK-LNPs/Rop increased over time, contributing to their significantly extended duration of action. Nonetheless, the results suggested that the duration of action of the two LNPs did not exceed 48 h; when the analgesic effect was exhausted, the paw withdrawal threshold and paw withdrawal latency returned to the level prior to the treatment, indicating that mice were re-suffering from severe neuropathic pain. Thus, there is an urgent need for long-term analgesia. Given that ADAM8 was persistently upregulated in the DRG within at least 28 days following SNI modeling (Fig. 3D), BK-LNPs/Rop is supposed to be suitable for long-term use for analgesia improvement. To prove it, BK-LNPs/Rop was further intravenously injected on the 14th and 21st days following SNI modeling. As expected, BK-LNPs/Rop significantly relieved mechanical allodynia and thermal hyperalgesia in the repeated injections (Fig. 4F–M). No drug resistance was observed in the repeated injections. Analgesic action for each drug formulation in the repeated injections was similar. Generally, BK-LNPs/Rop effectively relieved neuropathic pain in a single or repeated injections and particularly, showed superiority over LNPs/Rop in attenuating pain intensity.

Interestingly, Rop-free BK-LNPs also demonstrated a certain analgesic effect, which should be owing to its binding with upregulated ADAM8 in the DRG. Thus, it needs to be clarified that the improved analgesic effect of BK-LNPs/Rop compared with LNPs/Rop results from DRG-targeted delivery rather than a synergistic analgesic effect from BK-1361. This point was ascertained by the results that intrathecal injection of BK-LNPs/Rop did not produce an analgesic effect superior to LNPs/Rop (Fig. S8), due to the lack of DRG-targeting ability in this administration route (Fig. S4). Moreover, despite improved analgesic effect, BK-LNPs/Rop failed to extend the duration compared with LNPs/Rop. It is thus suggested that the analgesic effect of a pain nanomedicine should positively correlate with its dose, whilst its duration of action highly relies on the release kinetics of nanomedicine. In this sense, designing a pain nanomedicine with more sustained release profiles would be more beneficial to the duration of action, although it might impair the analgesic effect to some extent. This hypothesis can be partially identified by the following findings: 1) free Rop showed good analgesic effect in the short period following injection, as it directly acted on cells, but significantly short duration of action in comparison to LNPs/Rop and BK-LNPs/Rop (Fig. 4B–M and Fig. S7); 2) LNPs with more sustained release profiles showed extended duration of action compared with liposomes (Figure S3 and Figure S9).

The psychiatric disorders known to significantly affect quality of life are always present in long-term painful conditions, as a result of the fear of painful sensation. The two most common psychiatric comorbidities in neuropathic pain are anxiety and depression. In a study of 61 patients with neuropathic pain, the prevalence of anxiety and depression was up to 73.7 % and 65.6 %, respectively [51]. Both anxiety and depression are positively correlated with pain duration and intensity, and are reported to be often associated with poor response to neuropathic pain treatment [52]. Thus, the outcome of BK-LNPs/Rop in relieving anxiety and depression deserves attention. EPM and FST were conducted to evaluate anxiety- and depression-like behaviors of SNI mice, respectively, following repeated treatments. EPM device consists of a crossed platform in the middle with perpendicularly arranged open and closed arms. As anxiety is an emotional response to stress or danger, anxious mice are expected to spend less time in the unprotected open arms [53]. As shown in Fig. 4N and Fig. S10, SNI led to a significant decrease in relative time spent in the open arms, indicative of anxiety (∗*p* < 0.05, PBS vs sham). Free Rop as well as BK-LNPs treatment only slightly increased the time; LNPs/Rop and BK-LNPs/Rop treatment further increased the time and however, they showed no difference compared with the PBS group. BK-LNPs/Rop failed to significantly relieve anxiety, although a positive effect was found. Depression-like behavior was evaluated by FST. The behaviors of mice in FST can be swimming, struggling, or immobility, and the duration of immobility is the major indicator of depression. The results demonstrated that SNI also significantly decreased the duration of immobility (∗*p* < 0.05, PBS vs sham); while slightly decreasing the duration after the treatment with free Rop and BK-LNPs like that in EPM, LNPs/Rop and BK-LNPs/Rop treatment significantly decreased the duration for depression relief (∗*p* < 0.05, LNPs/Rop vs free Rop; ∗∗*p* < 0.01, LNPs/Rop vs PBS; ∗∗*p* < 0.01, BK-LNPs/Rop vs PBS) (Fig. 4O). EPM and FST results confirm that neuropathic pain induced by SNI can result in anxiety and depression, and there is a high correlation between anxiety and depression. Free Rop exerts a neglectable effect on relieving anxiety and depression, due to its short duration of action. Facilitated by extended duration of action, encapsulating Rop into LNPs improves the effect, particularly displaying a significant difference in the depression-like behavior evaluation. Moreover, there is no difference between LNPs/Rop and BK-LNPs/Rop, despite the better analgesic effect of the latter. Such an unexpected result might result from the fact that the outcome of LNPs/Rop has approached the plateau, which is comparable to the sham group. Together with the neglectable effect of free Rop, it is strongly suggested that the outcome of a pain nanomedicine in relieving psychiatric comorbidities is determined by its duration of action rather than its temporary intensity of action.

### 3.4 DRG-targeted analgesic delivery effectively relieves neuropathic pain in CINP

**Fig. 5:**
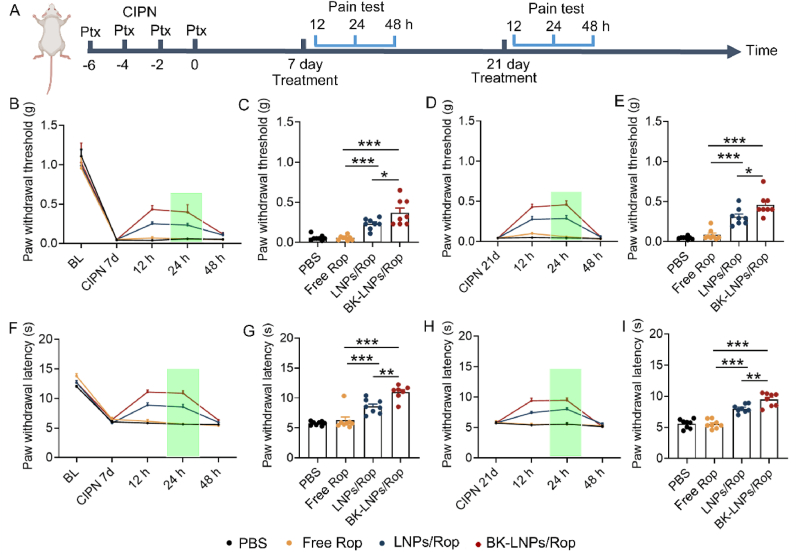
*In vivo* evaluation of neuropathic pain in CINP. (A) Protocol of CINP modeling and drug treatment for behavioral test. (B and D) Mechanical allodynia examination at 12 h, 24 h, and 48 h following the injection on the 7th and 21st days and (C and E) corresponding analysis at 24 h. (F and H) Thermal hyperalgesia examination at 12 h, 24 h, and 48 h following the injection on the 7th and 21st days and (G and I) corresponding analysis at 24 h. Mice received intravenous injection of PBS, free Rop, LNPs/Rop, and BK-LNPs/Rop at 3 mg Rop/kg body weight. BL data came from healthy mice before CINP modeling. Data were expressed as mean ± SEM (n = 8). ∗*p* < 0.05, ∗∗*p* < 0.01 and ∗∗∗*p* < 0.001.

CINP has no evidenced pain source and unfortunately, can persist for months to years. Currently, there is no efficacious drug for treatment or prevention and opioids are often recommended as palliatives, providing limited benefit accompanied with the risk of dependence. Encouraged by the involvement of ADAM8 in CINP and especially, its upregulation in the DRG, BK-LNPs also offer a targeted treatment alternative to CINP (Fig. 5A). Mechanical allodynia and thermal hyperalgesia profiles revealed that repeated intraperitoneal injections of Ptx led to sharply decreased paw withdrawal threshold and paw withdrawal latency, indicating the success of CINP modeling. In a single or repeated injections of different drug formulations, the analgesic effect of free Rop failed to last 12 h and LNPs/Rop significantly extended the duration of action for at least 24 h; BK-LNPs/Rop significantly improved the intensity of action (Fig. 5B–I). Together with the results in SNI, it was concluded that the pain manifestation of SNI and CIPN and the outcome of different Rop formulations in the two neuropathic pain models were in a high degree of resemblance; BK-LNPs/Rop effectively relieved neuropathic pain through DRG-targeted delivery, although the causes and mechanisms of the two models were different.

### 3.5 Safety assessment

It has to be admitted that opioids should be ideal drugs for DRG-targeted delivery through intravenous route, due to their satisfactory analgesic effect and applicability for intravenous use. However, their acquisition is legally restricted. Local anaesthetics with Rop, bupivacaine, and lidocaine as representatives are a group of medications that inhibit NaV channel and thereby block signal transduction in nerve fibers for pain relief. Inspired by their relatively satisfactory analgesic effect and better safety than opioids, local anaesthetics are the most studied analgesics involved in pain nanomedicine, offering notable advantages in various settings such as peripheral nerve blockade, obstetrics, and surgery [54]. Although local anaesthetics are always suggested for topical use, they can also be intravenously injected with manageable risk within safe dose, as revealed by some meta-analyses [55,56]. Combined with the finding that local anaesthetics can relieve neuropathic pain by acting the neurons in the DRG with the mechanisms beyond NaV channel blocking alone [57,58], they thus should be a suitable model drug to determine whether DRG-targeted analgesic accumulation mediated by BK-LNPs can improve the outcome. Rop, the same family with bupivacaine, shows comparable potency and reduced toxicity compared with bupivacaine as well as lidocaine and is finally chosen as the payload of BK-LNPs [59].

**Fig. 6:**
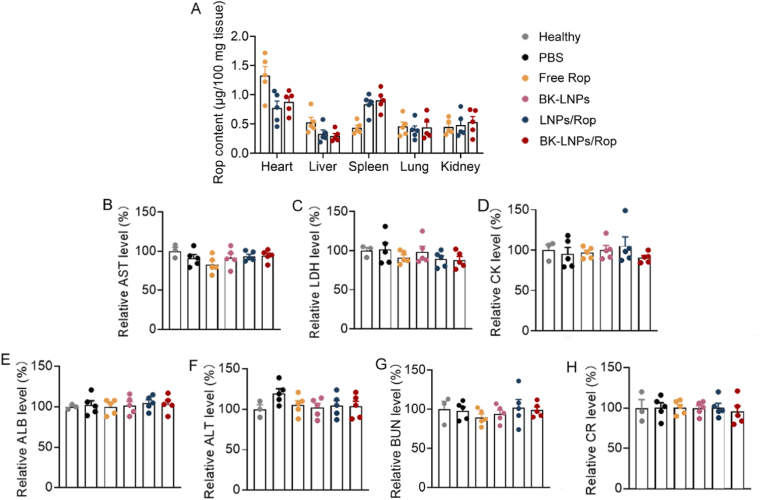
Safety assessment. (A) The tissue distribution of free Rop, LNPs/Rop, and BK-LNPs/Rop in the heart, brain, liver, spleen, lung, and kidney. The distribution in the brain was not detected, limited by detection sensitivity. SNI mice received intravenous injection once and the distribution was determined at 6 h post-injection. (B–H) Hematologic study for AST, LDH, CK, ALB, ALT, BUN and CR analyses reflecting heart, liver, and kidney functions. SNI mice received repeated intravenous injections. The dose of Rop was 3 mg/kg. Data were expressed as mean ± SEM (n = 3–5).

Life-threatening systemic adverse events of Rop are very rare (<1/1000), which is always associated with overdosing, especially when given in a short period. On the other hand, although the optimal intravenous treatment regimen of local anaesthetics remains unestablished, as a result of high individual heterogeneity, 1.5–3 mg/kg is reported to be a safe dose for patients, while achieving therapeutic outcome [60,61]. The dose of Rop was herein fixed at 3 mg/kg. Following intravenous injection, a drug will circulate in the blood and be then widely accumulated into various tissues for metabolism. For local anaesthetics, their high peak concentration in the plasma and the accumulation in the heart and brain as well as the low tolerance of these two tissues to specific drug metabolisms pathways are the causes of the cardiovascular system (CVS) and central nervous system (CNS) toxicities [62]. Moreover, Rop is extensively metabolized in the liver. Thereby, the plasma drug concentration and distribution of Rop in different formulations in the heart, brain, and liver as well as some other main tissues are our concern, which was carried out in SNI mice receiving intravenous injection once. As shown in Table S3, free Rop underwent a rapid metabolism with a high plasma drug concentration at 1 min post-injection sharply decreasing to be undetectable at 2 h post-injection. In comparison, although the plasma drug concentration of LNPs/Rop and BK-LNPs at 1 min and 10 min post-injection was slightly lower than that of free Rop, it maintained a detectable level at 6 h post-injection. No difference was observed between LNPs/Rop and BK-LNPs/Rop. Plasma drug concentration profiles suggested that while improving pharmacokinetics to ensure extended duration of action, LNPs encapsulation potentially reduced peak plasma drug concentration, indicative of better safety. Tissue distribution demonstrated that LNPs altered the metabolic pathway of Rop and reduced its accumulation in the heart and liver (Fig. 6A). Particularly, the reduced accumulation in the heart signified a reduced risk of CVS toxicity. Moreover, surface BK-1361 functionalization did not alter the distribution of LNPs, according well with the fact that the expression of ADAM8 in normal tissues was inherently low [63]. The distribution of all formulations in the brain was not detected, owing to low drug distribution and limited detection sensitivity by HPLC.

Systemic adverse events were concurrently assessed in SNI mice receiving repeated treatments with the protocols illustrated in Fig. 4A. CVS and CNS toxicities are the most common adverse events of local anaesthetics and present with a multitude of symptoms in patients (seizure, arrhythmia, acute respiratory failure, cardiac arrest, convulsion, etc.), depending on dose. Throughout the experimental period, no such apparent CVS and CNS toxicities were observed and no death occurred. Although experiencing severe neuropathic pain, SNI mice even showed weight gain (data not shown), also indicating that they did not suffer latent systemic toxicities. At the end of the experiment, serum AST, LDH, CK, ALB, ALT, BUN, and CR levels were analyzed. Fig. 6B–D demonstrated that treatments with free Rop, LNPs/Rop, BK-LNPs/Rop as well as BK-LNPs did not lead to the abnormalities of AST, LDH and CK serving as heart dysfunction indicators [64]. Similarly, no abnormalities were observed in ALB, ALT, BUN, and CR that reflected liver and kidney functions (Fig. 6E–H). Additionally, H&E staining revealed that no histopathological characteristics were observed in the heart, brain, liver, spleen, lung, and kidney in all groups (Fig. S11). Generally, within the safe dose, intravenously injected free Rop did not cause biochemical and histomorphological abnormalities as compared to the healthy group. LNPs/Rop decreased the peak plasma drug concentration, which would certainly benefit safety. Furthermore, LNPs/Rop prolonged Rop circulation in the blood and encouraged Rop accumulation in some tissues; however, it would not harm safety, which was extensively verified by approved cancer nanomedicines. In fact, a clinically effective technique to reverse systemic adverse events of local anaesthetics is intravenous lipid emulsion 20 %. Lipid emulsion is made of soybean oil, egg phospholipid, and glycerol, offering beneficial effect by acting as a nanocarrier to reduce plasma drug concentration and a lipid provider to influence drug metabolism [62]. In terms of the component and structure, LNPs/Rop are safe. Importantly, BK-LNPs/Rop did not promote the accumulation in normal tissues, due to inherently low expression of ADAM8, and thus exhibited good safety analogous to LNPs/Rop. On the other hand, to achieve an analgesic effect equal to LNPs/Rop, less BK-LNPs/Rop is demanded, which can reduce the risk of systemic adverse events.

## 4 Conclusion

In summary, a DRG-targeted analgesic delivery strategy using functionalized LNPs was reported to treat neuropathic pain by blocking pain signal transduction from the periphery to central nervous system. Results revealed that plain LNPs had a more sustained release manner than liposomes, benefiting the duration of action and thus showing therapeutic superiority. Once functionalized with the targeting peptide, the LNPs enabled analgesic accumulation in the DRG through the targeting affinity between the peptide and ADAM8 upregulated in the DRG, induced by SNI and CIPN, two neuropathic pain models with distinct mechanisms. As a result, the LNPs significantly improved the intensity of action, effectively relieving pain and its-related psychiatric comorbidities. Together with their good safety, the LNPs emerged as a safe, effective, and universally applicable analgesic delivery system, beyond delivering local anaesthetics, to treat neuropathic pain.

## CRediT authorship contribution statement

**Jiajia Sun:** Writing – original draft, Validation, Methodology, Investigation, Formal analysis. **Jia Gu:** Writing – original draft, Validation, Investigation, Formal analysis. **Yan Ding:** Methodology, Investigation, Formal analysis. **Xinyi Tu:** Investigation. **Xiaohui Cai:** Writing – review & editing, Validation, Methodology, Funding acquisition. **Baochun Jiang:** Writing – review & editing, Validation, Software, Funding acquisition, Conceptualization. **Zhongping Chen:** Writing – review & editing, Validation, Supervision, Methodology, Funding acquisition, Data curation, Conceptualization.

## Declaration of competing interest

The authors declare that they have no known competing financial interests.

## Data Availability

Data will be made available on request.
