# Supplementary material for: Dorsal root ganglion-targeted analgesic delivery for effective relief of neuropathic pain
Source: Mater Today Bio. 2025 Jun 26;33:102025. doi: 10.1016/j.mtbio.2025.102025 (PMC12272124; doi:10.1016/j.mtbio.2025.102025)
Supplement: Multimedia component 1 [file mmc1.docx]

Supporting materials

Dorsal root ganglion-targeted analgesic delivery for effective relief of neuropathic pain

Jiajia Sun ^1,a^, Jia Gu ^1,a^, Yan Ding ^1,a^, Xinyi Tu ^a^, Xiaohui Cai ^c,^*, Baochun Jiang ^b,d,^*, and Zhongping Chen ^a,^*

^a^ Institute of Special Environmental Medicine, Nantong University, Nantong, People's Republic of China

^b^ Department of Anesthesiology, the Second Affiliated Hospital, Zhejiang University School of Medicine, Hangzhou, People's Republic of China

^c^ Department of Hematology, the Third Affiliated Hospital of Nanjing Medical University, Changzhou, People's Republic of China

^d^ Zhejiang Key Laboratory of Pain Perception and Neuromodulation, Hangzhou, People's Republic of China

^*^Corresponding author: sosocaixiaohui@163.com (Xiaohui Cai); [jiangbaochun@zju.edu.cn](mailto:jiangbaochun@zju.edu.cn) (Baochun Jiang); [chenzp@ntu.edu.cn](mailto:chenzp@ntu.edu.cn) (Zhongping Chen). Tel: +86513-55003378; Fax: +86513-55003378

^1^These authors contributed equally to this work

Size, morphology, PDI, and zeta potential of LNPs

TEM imaging was performed to directly observe the size and morphology of LNPs. Sample solution was dropped on a copper grid and then negatively stained with 1% phosphotungstic acid solution (w/v). TEM imaging was operated at 80.0 kV. Hydrodynamic size, PDI, and zeta potential of LNPs were determined by DLS technology. Sample solution was diluted to a count rate of 100-300 kcps before the analysis and at least triplicate experiments were carried out.

Synthesis of Rop-loaded liposomes (Liposomes/Rop)

Liposomes/Rop were synthesized by classic thin film evaporation and ultrasonic hydration for stability comparison with LNPs/Rop. DOPE, cholesterol, DSPE-PEG, and Rop in the same ratio with that used to synthesize LNPs/Rop were dissolved in dichloromethane in a round-bottomed flask. After rotary evaporation under vacuum to remove the organic solvent, the lipid film was hydrated with water under bath sonication, followed by probe sonication, generating liposomes/Rop.

Rop quantification, entrapment efficiency, loading capacity, and *in vitro* release by HPLC

Rop was quantified by HPLC. Twenty microliters of Rop dissolved in methanol with different concentrations (1, 5, 10, 20, and 40 μg/mL) were injected into HPLC for analysis. HPLC conditions were as follows: chromatographic column, ultimate^®^AQ-C_18_; mobile phase, 0.02 M KH_2_PO_4_/acetonitrile (65:35, v/v); flow rate, 1 mL/min; column temperature, 30℃; injection volume, 20 μL; detection wavelength, 210 nm. At these conditions, Rop could be eluted at 5.3 min with a sharp peak. A standard curve was obtained by plotting Rop peak area against its concentration (y=57624x-2655.8, R^2^=0.999).

To determine drug entrapment efficiency and drug loading capacity, 0.1 mL of Rop -loaded LNPs (LNP/Rop and BK-LNPs/Rop) was treated with 1.9 mL of methanol and then sonicated to completely destroy the structure of LNPs. After centrifugation at 8000 rpm for 10 min, the supernatant was collected and then filtered with 0.45 μm organic membrane. Loaded Rop in filtrate was determined by HPLC. Drug entrapment efficiency was defined as loaded Rop relative to initially fed Rop (w/w); drug loading capacity was defined as loaded Rop relative to loaded Rop plus total lipids (w/w).

In another experiment to assess *in vitro* drug release of Rop from LNPs, 5 mg of lyophilized LNPs powder (LNP/Rop and BK-LNPs/Rop) dissolved in 10 mL of PBS (pH = 7.4) in a 50 mL EP tube was placed on a shaking table with gentle shaking (170 rpm, 37℃). At predetermined timepoints, 100 μL of PBS solution was withdrawn. Following centrifugation at 15000 rpm for 10 min, released Rop in supernatant was collected and then treated with 1.9 mL of methanol for HPLC analysis according to the protocol described above. *In vitro* drug release was assessed by plotting cumulative released percentage of Rop over time. For free Rop, its methanol solution was dispersed into PBS (pH = 7.4) for *in vitro* release assessment.

Plasma drug concentration and tissue distribution of Rop

Blood samples were treated with a mixed solution of methanol and chloroform (1/3, v/v), followed by sonication, to extract Rop. After centrifugation at 8000 rpm for 10 min, extracted Rop in organic layer was collected. The organic phase was evaporated and the samples were redissolved in methanol, followed by centrifugation at 8000 rpm for another 10 min, to completely precipitate tissue protein. Finally, the methanol layer containing Rop was collected and filtered with 0.45 μm filter for HPLC analysis with the above-described HPLC conditions. Tissue samples were dispersed in PBS (pH = 7.4) and underwent vigorous smash using a homogenizer before being treated with the mixed solution of methanol and chloroform

Table S1: Hydrodynamic size, PDI, and zeta potential(mV)of LNPs and BK-LNPs with or without RhB and Rop loading, determined by DLS. Data were presented as mean ± SD (n = 3).

| Formulation | Hydrodynamic size (nm) | PDI | Zeta potential(mV) |
| --- | --- | --- | --- |
| LNPs | 141 ± 2 | 0.28 ± 0.04 | -23.0 ± 0.8 |
| BK-LNPs | 138 ± 2 | 0.24 ± 0.04 | -24.5 ± 0.7 |
| LNPs/RhB | 162 ± 4 | 0.23 ± 0.02 | -26.1 ± 0.8 |
| BK-LNPs/RhB | 159 ± 2 | 0.26 ± 0.01 | -27.3 ± 0.9 |
| LNPs/Rop | 152 ± 1 | 0.21 ± 0.02 | -25.9 ± 2.4 |
| BK-LNPs/Rop | 143 ± 1 | 0.27 ± 0.02 | -25.2 ± 4.0 |

Table S2: Drug entrapment efficiency and loading capacity of LNPs/Rop and BK-LNPs/Rop. Data were presented as mean ± SD (n = 3).

| Formulation | Entrapment efficiency (%) | Loading capacity (%) |
| --- | --- | --- |
| LNPs/Rop | 85 ± 3 | 17.5 ± 0.8 |
| BK-LNPs/Rop | 89 ± 5 | 18.2 ± 1.1 |

Table S3 Plasma drug concentration of free Rop, LNPs/Rop, and BK-LNPs/Rop at 10 min, 1 h, 2 h, and 6 h post-injection. SNI mice received intravenous injection of free Rop, LNPs/Rop, and BK-LNPs/Rop at a dose of 3 mg/kg once. Data were expressed as mean ± SEM (n =5). n.d. = not detectable. It was noted that Rop content below 1 μg/mL in blood sample was not suitable for quantification by HPLC and thus considered not detectable.

|  | Plasma drug concentration (μg/mL) over time | | | | |
| --- | --- | --- | --- | --- | --- |
|  | 1 min | 10 min | 1 h | 2 h | 6 h |
| Free Rop | 5.62 ± 1.43 | 4.93 ± 0.98 | 1.78 ± 0.58 | n.d. | n.d. |
| LNPs/Rop | 4.90 ± 1.21 | 4.04 ± 1.03 | 2.74 ± 0.67 | 2.12 ± 0.56 | 1.32 ± 0.35 |
| BK-LNPs/Rop | 4.81 ± 0.72 | 3.93 ± 0.86 | 2.82 ± 0.75 | 2.29 ± 0.92 | 1.55 ± 0.43 |


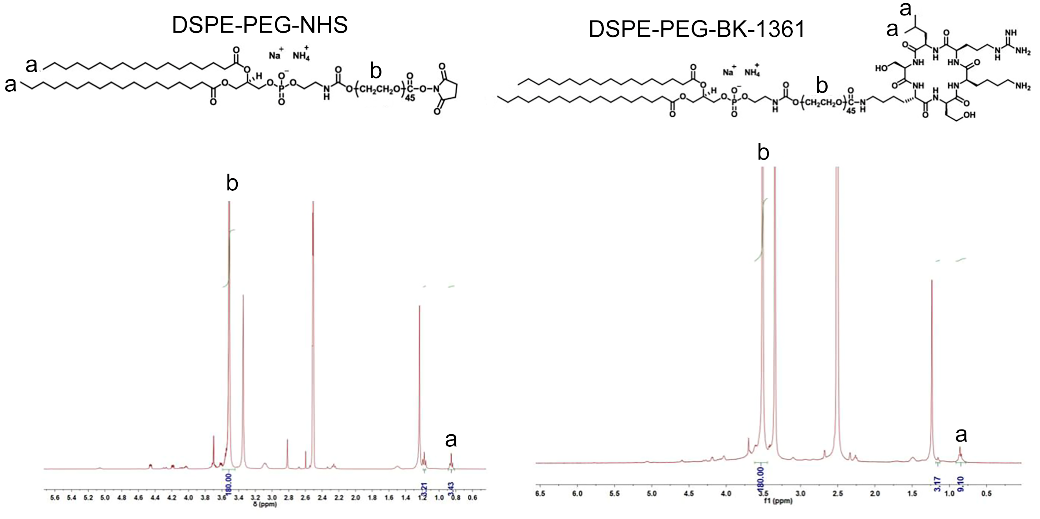


Figure S1: ^1^H NMR spectra of DSPE-PEG-NHS and DSPE-PEG-NHS-BK-1361.


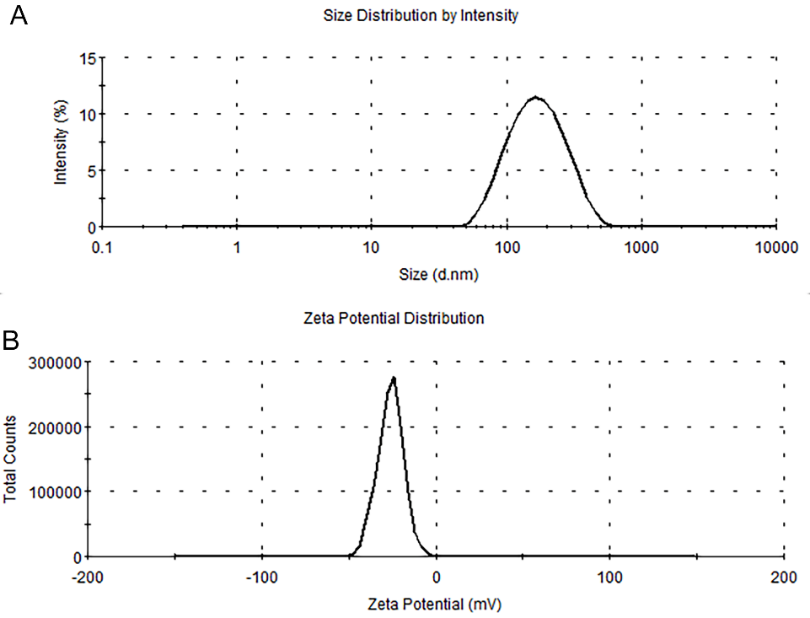


Figure S2: (A) Hydrodynamic size and (B) zeta potential of BK-LNPs/Rop, acquired from DLS.


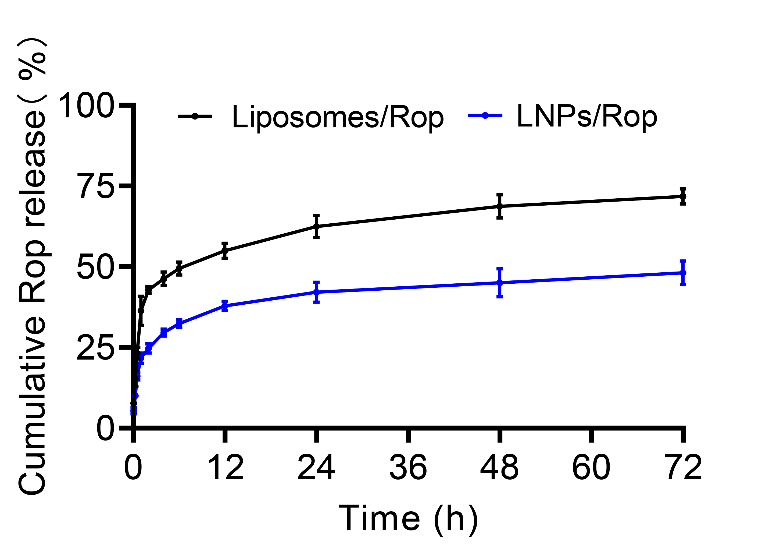


Figure S3: Cumulative drug release of LNPs/Rop and Liposomes/Rop within 72 h. Data were expressed as mean ± SD (n = 3).


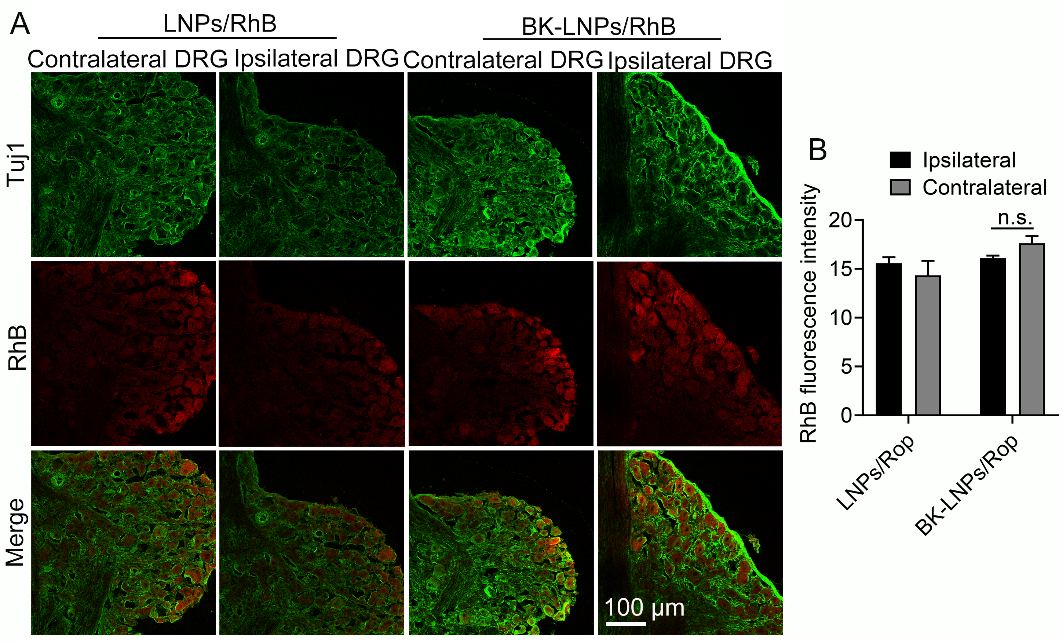


Figure S4: (A) CLSM imaging for the distribution of BK-LNPs and LNPs in the bilateral DRGs ipsilateral and contralateral to SNI surgical site and (B) corresponding quantitative analysis of RhB fluorescence. Mice were intrathecally injected with LNP/RhB and BK-LNPs/RhB and at 6 h post-injection, mice were euthanized for analysis. Data were expressed as mean ± SEM (n = 3).


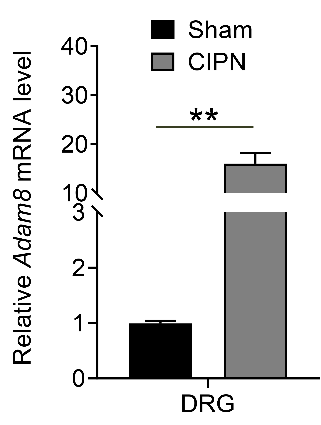


Figure S5: Relative *Adam8* mRNA expression level in the DRGs on the 21st day following CIPN modeling. Data were expressed as mean ± SEM (n = 3). ***p* < 0.01


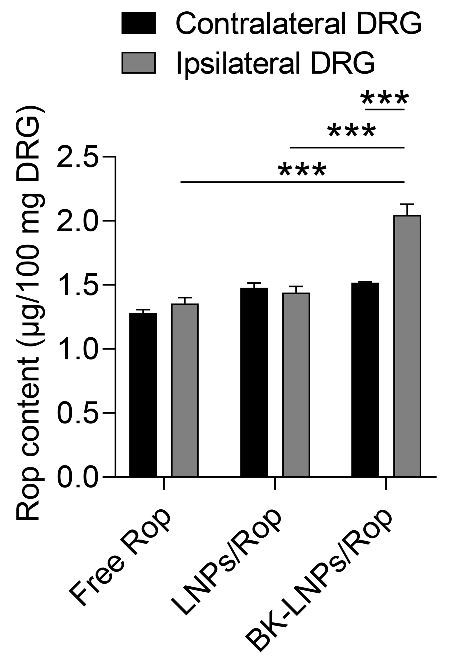


Figure S6: Drug distribution of free Rop, LNPs/Rop, and BK-LNPs/Rop in the DRGs contralateral and ipsilateral to SNI surgical site at 2 h post-injection. Mice received intravenous injection of free Rop, LNPs/Rop, and BK-LNPs/Rop at 3 mg Rop/kg body weight. Data were expressed as mean ± SEM (n = 3). ****p* < 0.001


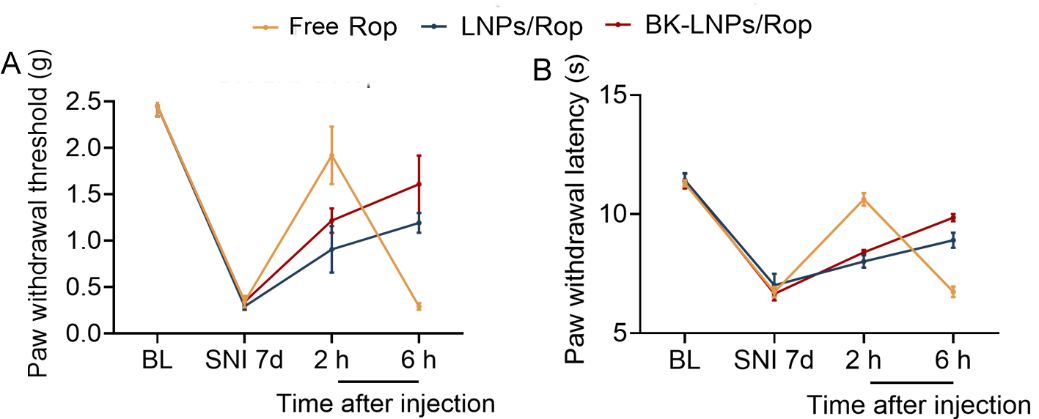


Figure S7 *In vivo* evaluation of neuropathic pain following intravenous injection in SNI mice. (A) Mechanical allodynia and (B) thermal hyperalgesia examinations at 2 h and 6 h post-injection. Mice received intravenous injection of free Rop, LNPs/Rop, and BK-LNPs/Rop at 3 mg Rop/kg body weight. BL data came from healthy mice before SNI modeling. Data were expressed as mean ± SEM (n = 3).


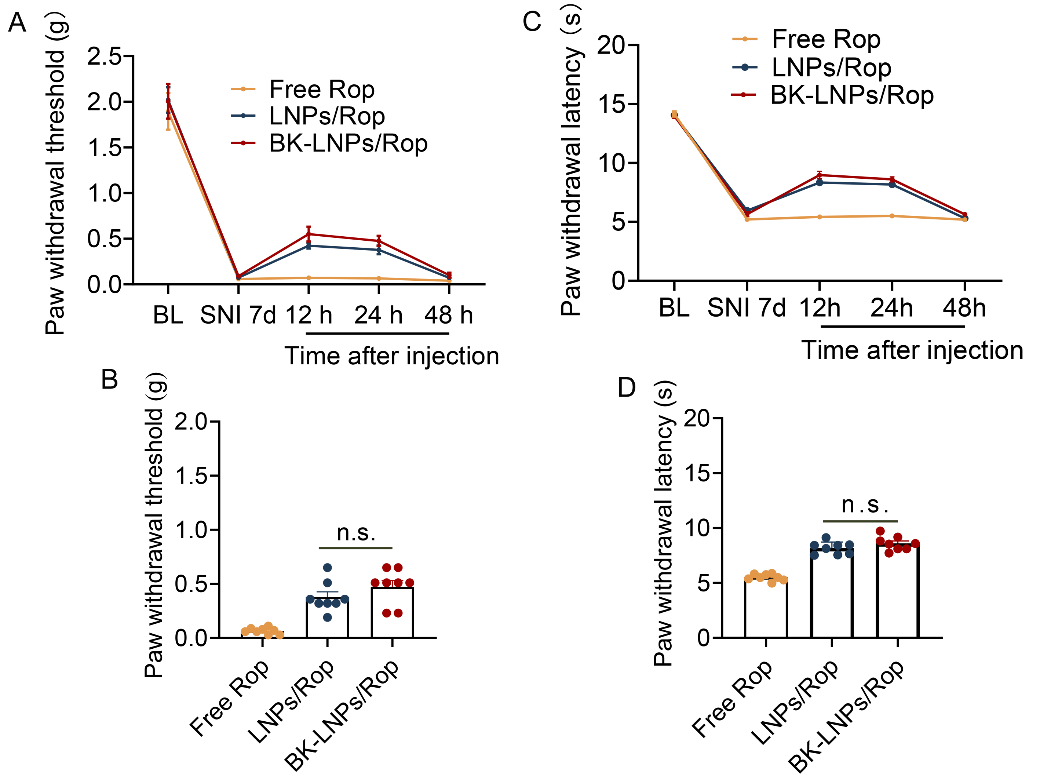


Figure S8: *In vivo* evaluation of neuropathic pain following intrathecal injection in SNI. (A) Mechanical allodynia examination at 12 h, 24 h, and 48 h following the injection on the 7th day and (C) corresponding analysis at 24 h following the injection. (B) Thermal hyperalgesia examination at 12 h, 24 h, and 48 h following the injection on the 7th day and (D) corresponding analysis at 24 h following the injection. Mice received intrathecal injection of free Rop, LNPs/Rop, and BK-LNPs/Rop at 1 mg Rop/kg body weight. BL data came from healthy mice before SNI modeling. Data were expressed as mean ± SEM (n = 8). n.s. = not significant.


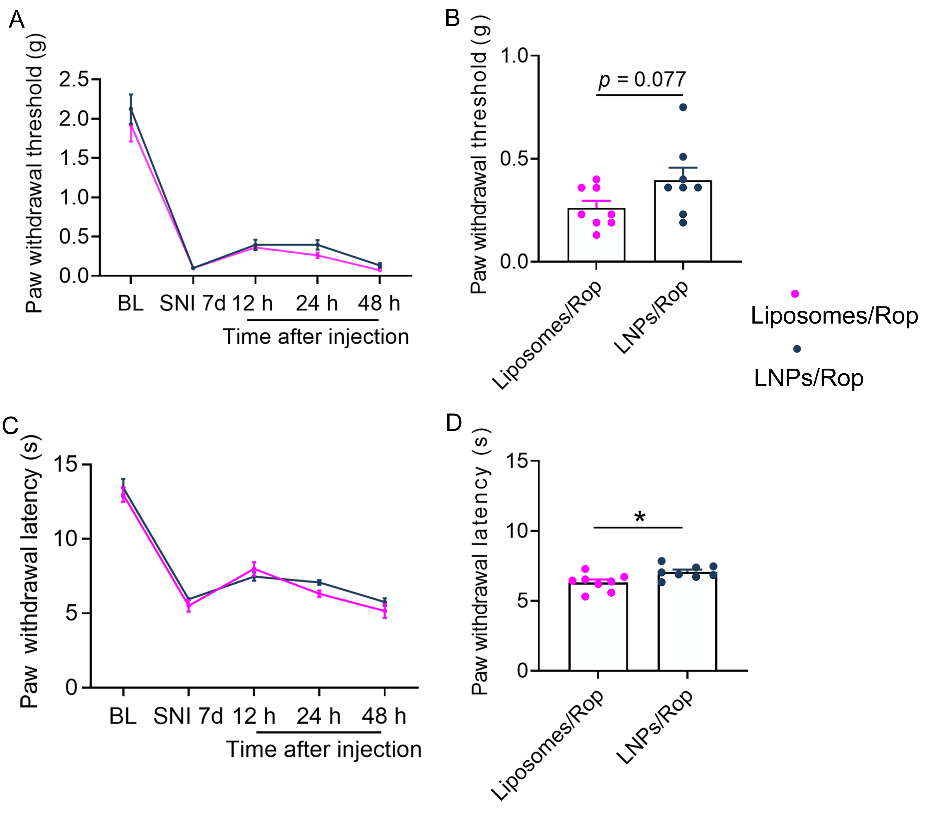


Figure S9: Analgesic effect comparison of liposomes/Rop and LNPs/Rop in SNI. (A) Mechanical allodynia examination at 12 h, 24 h, and 48 h following the injection on the 7th day and (B) corresponding analysis at 24 h following the injection. (C) Thermal hyperalgesia examination at 12 h, 24 h, and 48 h following the injection on the 7th day and (D) corresponding analysis at 24 h following the injection. Mice received intravenous injection of liposomes/Rop and LNPs/Rop at 3 mg Rop/kg body weight. BL data came from healthy mice before SNI modeling. Data were expressed as mean ± SEM (n = 8). **p* < 0.05.


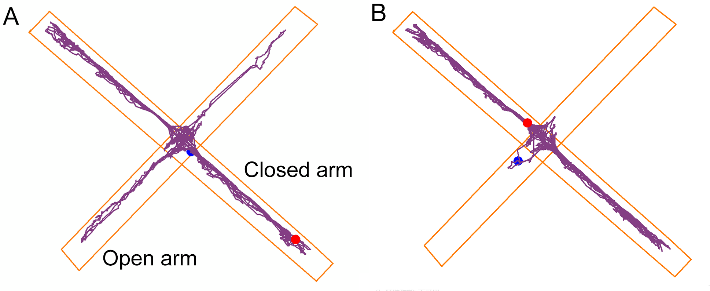


Figure S10: Representative movement trajectory of mice (A) without and (B) with anxiety-like emotion in the EPM test. Anxious mice spend more time in the open arms than non-anxious mice. Black and white dotes indicate track start and end, respectively.


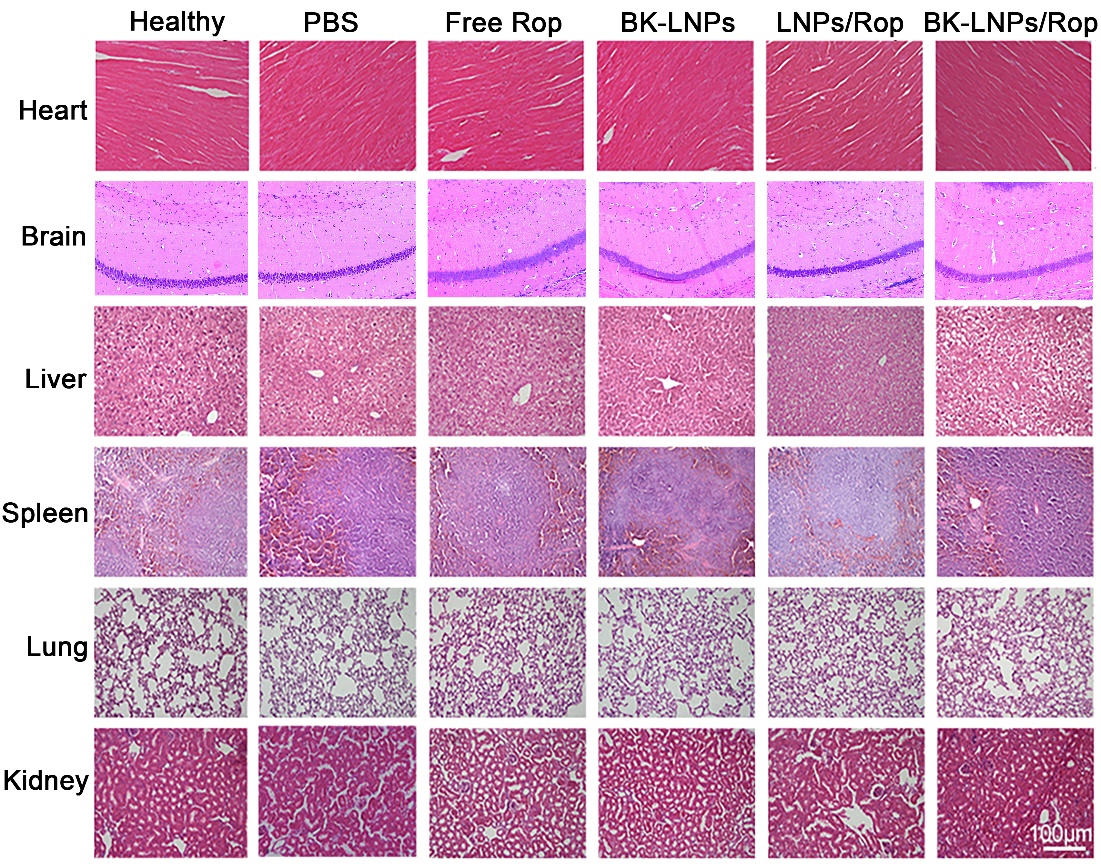


Figure S11: H&E staining of the heart, brain, liver, spleen, lung, and kidney in SNI receiving repeated treatments.
